# Effect of illumination level [18F]FDG-PET brain uptake in free moving mice

**DOI:** 10.1371/journal.pone.0251454

**Published:** 2021-05-13

**Authors:** Alexandra de Francisco, Yolanda Sierra-Palomares, María Felipe, Daniel Calle, Manuel Desco, Lorena Cussó

**Affiliations:** 1 Instituto de Investigación Sanitaria Gregorio Marañón, Madrid, Spain; 2 Unidad de Imagen Avanzada, Centro Nacional de Investigaciones Cardiovasculares (CNIC), Madrid, Spain; 3 Centro de Investigación Biomédica en Red de Salud Mental (CIBERSAM), Madrid, Spain; 4 Departamento de Bioingeniería e Ingeniería Aeroespacial, Universidad Carlos III de Madrid, Madrid, Spain; IRCCS Ospedale Policlinico San Martino, Genova, ITALY

## Abstract

In both clinical and preclinical scenarios, 2-deoxy-2[18F]fluoro-D-glucose ([18F]FDG) is the radiotracer most widely used to study brain glucose metabolism with positron emission tomography (PET). In clinical practice, there is a worldwide standardized protocol for preparing patients for [18F]FDG-PET studies, which specifies the room lighting. However, this standard is typically not observed in the preclinical field, although it is well known that animal handling affects the biodistribution of [18F]FDG. **The present study aimed to evaluate the effect of ambient lighting on brain [18F]FDG uptake in mice**. Two [18F]FDG-PET studies were performed on each animal, one in light and one in dark conditions. Thermal video recordings were acquired to analyse animal motor activity in both conditions. [18F]FDG-PET images were analysed with the Statistical Parametric Mapping method. The results showed that [18F]FDG uptake is higher in darkness than in light condition in mouse nucleus accumbens, hippocampus, midbrain, hindbrain, and cerebellum. The SPM analysis also showed an interaction between the illumination condition and the sex of the animal. Mouse activity was significantly different (p = 0.01) between light conditions (632 ± 215 s of movement) and dark conditions (989 ± 200 s), without significant effect of sex (p = 0.416). We concluded that room illumination conditions during [18F]FDG uptake in mice affected the brain [18F]FDG biodistribution. Therefore, we highlight the importance to control this factor to ensure more reliable and reproducible mouse brain [18F]FDG-PET results.

## Introduction

Positron emission tomography (PET) is a non-invasive imaging technique that enables in vivo studies of many physiological and pathological processes, such as Alzheimer disease [1], cancer [2], or infectious diseases [3]. In both clinical and preclinical settings, the radiotracer most widely used to study brain glucose metabolism with PET is 2-deoxy-2[18F]fluoro-D-glucose ([18F]FDG), due to its numerous clinical indications [4] and its high sensitivity and availability.

In clinical practice, there is a worldwide standardized protocol for preparing patients for [18F]FDG-PET studies [5]. This protocol includes the control of many factors in the days prior to and during imaging. For example, during the uptake period, factors such as noise, activity, illumination, and temperature can affect the [18F]FDG biodistribution in patients. These factors are even more important in neurological examinations, where the patient must be relaxed, in a room with dim light and low noise, to maintain normal brain metabolism during [18F]FDG uptake [5, 6].

In contrast, preclinical research with rodents lacks standardization of [18F]FDG-PET imaging, even in neurological applications. Several studies have shown that many factors, including anaesthesia, fasting, the route of radiotracer delivery, ambient temperature, etc., can affect the biodistribution of [18F]FDG in rodents [7–9]. Nevertheless, very few studies have described illumination conditions during brain [18F]FDG uptake in animal studies. Recent efforts that focused on standardizing protocols for [18F]FDG-PET studies in rodents [9] have missed information about room illumination. Therefore, the present study aimed to evaluate the effect of room illumination on brain [18F]FDG uptake in mice.

## Materials and methods

### Design and animals

This study included 39 11-week-old wild type C57BL/6J mice from the Animal Facility of the Centro Nacional de Investigaciones Cardiovasculares (CNIC). Mice were housed in a number of 4–5 per cage under a 12-h light–dark cycle at 23° ± 1°C and 50 ± 5% humidity, and were allowed access to food and water ad libitum. Before the imaging studies, mice were fasted for 8-h with *ad libitum* water. Twenty-four of the mice (50% females) underwent two [18F]FDG-PET studies under different lighting conditions (lightness and darkness) during uptake, with a 48-h interval between the two imaging studies. Before the first PET-CT study animals were randomized into two groups, half of them receiving light and the other half dark during the [18F]FDG uptake period. 48 hours later the imaging study was repeated reversing the light/dark condition to which the animals were subjected. In both imaging sessions the animals were analyzed at the same time of day, in order to reduce possible interference of the circadian rhythm [10], and the room temperature was kept at 23° ± 1°C.

**Fig 1:**
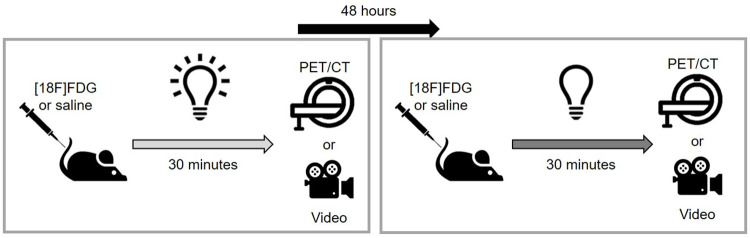
Experimental design. Awake animals were exposed to light or darkness in two different days during 33.6 ± 2.7 min before the PET/CT acquisition (n = 24) or video recording (n = 15). Before the illumination condition animals received an intravenous administration of [18F]FDG or saline.

The other 15 animals (8 males, 7 females) were used to analyse motor activity under light and dark conditions, by recording videos with a thermal camera (Fig 1), in a room temperature of 23° ± 1°C.

### Brain imaging studies

Brain PET studies were carried out with a SuperArgus small-animal PET-computed tomography (CT) scanner (SEDECAL, Madrid). Mice received 18 ± 1 MBq [18F]FDG by tail vein administration under inhaled anaesthesia (3% sevoflurane in 100% oxygen). Then animals were kept awaken and freely moving during the uptake period (33.6 ± 2.7 min), under either light (780 lx, white fluorescent light source OSRAM DULUX L 55W/840) or dark (0 lx) ambient conditions. After the uptake period, animals were again anesthetized (3% sevoflurane in 100% oxygen) for the PET-CT data acquisition (30 min). The respiration rate of the anesthetized animals was permanently monitored during the scan period using a small animal dedicated equipment (VisionPet, RGB). We used a 3-dimensional, ordered subset expectation maximization (3D-OSEM) reconstruction, with 16 subsets and 3 iterations. The voxel size of the reconstructed images was 0.388 mm in the transaxial plane and 0.775 mm in the axial plane. After the PET scan, a CT scan was performed with an X-ray beam current of 340 μA and a tube voltage of 40 kVp. We reconstructed the images with the Feldkamp, Davis, and Kres (FDK) algorithm [11].

### PET data

PET studies postprocessing was performed following protocols previously described by our group [12, 13]. PET images were spatially co-registered by rigid transformation to a randomly selected reference CT scan. Firstly, whole brain uptake was measured by defining a ROI covering the entire brain on the CT images and then obtaining the brain SUVmean from the same ROI in PET study. Then PET scans were normalized to global mean brain intensity and smoothed with a Gaussian kernel of 0.775 x 0.775 x 1.55 mm full-width at half-maximum (FWHM). A brain mask was segmented from a T2-weighted magnetic resonance image (MRI) acquired with a Biospin 7T (Bruker, Germany) with the following acquisition parameters: TR/TE = 4600/65 ms, slice thickness = 0.5 mm, matrix size 192 x 192, and field of view 15 x 15 mm. The MRI was also co-registered to the reference CT scan; then, the mask was applied to all PETs to exclude voxels outside the brain. We performed a voxel-wise statistical analysis of PET data with two dichotomous factors: the illumination condition (light/dark) and the animal sex (male/female), with the SPM method. We also performed a flexible factorial test, provided in the SPM12 software platform(http://www.fil.ion.ucl.ac.uk/spm/software/spm12/). This test included the interaction between the light condition and the sex of the animal. The significance threshold was p<0.05 and the cluster size was k = 25. The voxel size of the maps generated in the SPM analysis is the same than that of the original PET images. SPM maps were superposed to the acquired MRI scan to have an anatomical template. Data are reported as significance maps together with regional effect size in %.

### Animal activity

We studied motor activity in 15 animals (8 males, 7 females) by recording 30-min videos with a thermal camera (ThermaCAM SC2000 PAL, Flir Systems), under light (780 lx) or dark (0 lx) conditions. The two videos were recorded with a 48-h interval between videos. These animals were subjected to the same pre-imaging animal handling by anaesthetizing mice with inhalation anaesthesia (3% sevoflurane in 100% oxygen), then administering an intravenous injection of 0.2 ml physiological saline solution (0.9% NaCl, B Braun Medical). The recorded videos were analysed by an expert, who timed the periods of animal activity (expressed in seconds). We defined activity as animal movement over the cage floor or its grid. The first 2-min of the video were discarded, because they corresponded to recovery from the inhaled anaesthesia. An ANOVA linear mixed model was used to analyse the differences in motor activity between light and dark environments (IBM SPSS Statistics 20). Sex, the illumination condition, and the interaction between the two were fixed effects, and the individual mouse was the repeated factor. Data are expressed as the mean ± standard deviation.

### Ethics

Animals were housed in the animal facility of the Hospital General Universitario Gregorio Marañón (HGUGM), Madrid, Spain (authorization# ES280790000087). All animal procedures conformed to the EU Directive 2010/63EU and national regulations (RD 53/2013). All animal procedures were approved by the HGUGM Animal Experimentation Ethics Committee and by the Animal Protection Board of the Comunidad Autónoma de Madrid (PROEX 227/14).

## Results

### PET data analysis

**Fig 2:**
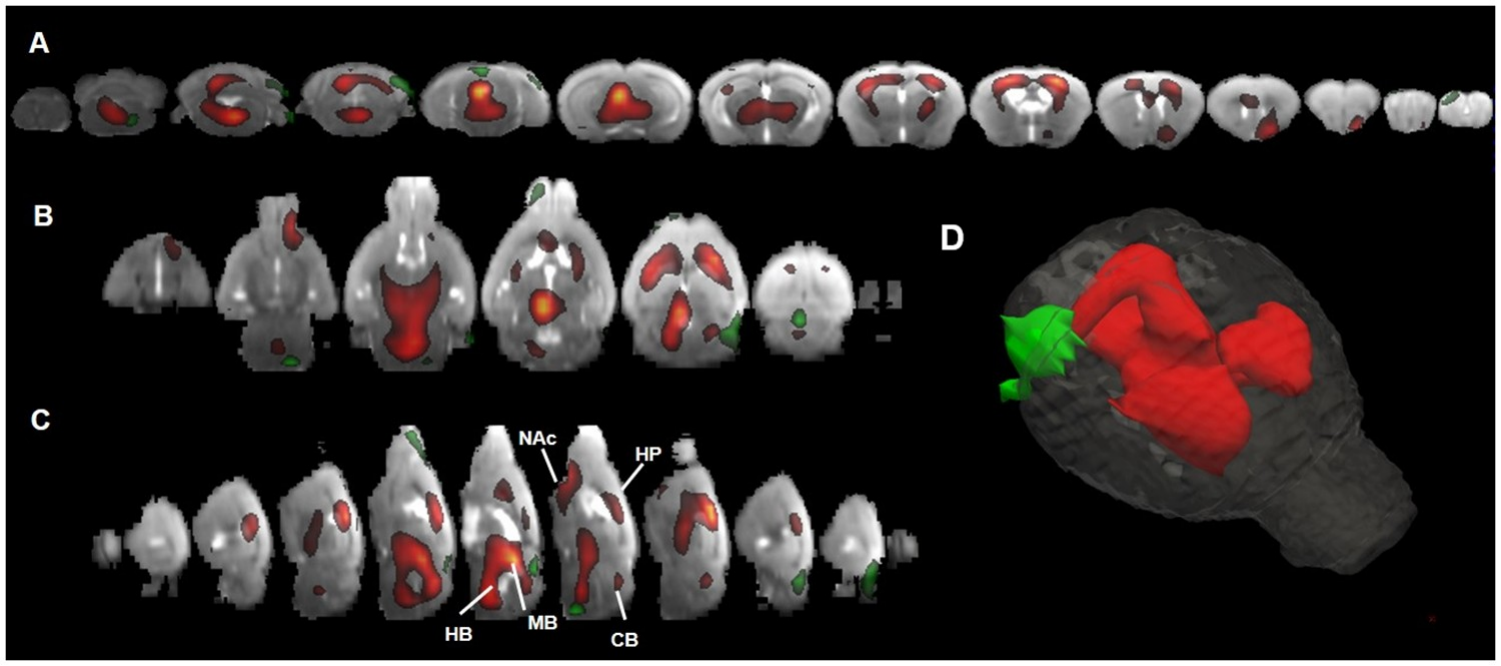
SPM results overlapped to an MRI scan template. The results are represented in axial (A), coronal (B) and sagittal (C) planes (p<0.05, t-test, k = 25 neighbor voxels). (D) 3D-render of the results. Red color represents the areas with higher [18F]FDG uptake in darkness while green color represents the interaction area between illumination conditions and gender. NAc: nucleus accumbes; HP: hippocampus; MB: midbrain; HB: hidbrain; CB: cerebellum.

No differences were found in the brain uptake (SUVmean) between illumination conditions (light 2.1 ± 0.5 vs. darkness 2.2 ± 0.6, p = 0.747) or sex (males 2.3 ± 0.4 vs. female 2.0 ± 0.7, p = 0.143), thus justifying the use of global mean scaling for intensity normalisation. The Statistical Parametric Mapping (SPM) results showed an increase in the [18F]FDG uptake that corresponded to a volume of 600 voxels (70 mm^3^), when the animals were in dark compared to light conditions (Fig 2A–2D, red). This volume corresponded to brain activity increases in the nucleus accumbens (+4.3%), hippocampus (+2.6%), midbrain (+2%), hindbrain (+3.3%), and cerebellum (+2.4%). The analysis also showed an interaction between the illumination condition and the sex of the animal. The difference between sexes in dark conditions was 40–50 voxels (5–6 mm^3^); this volume was located in the olfactory bulb, midbrain, hindbrain, and cerebellum (Fig 2D, green).

### Animal activity analysis

**Fig 3:**
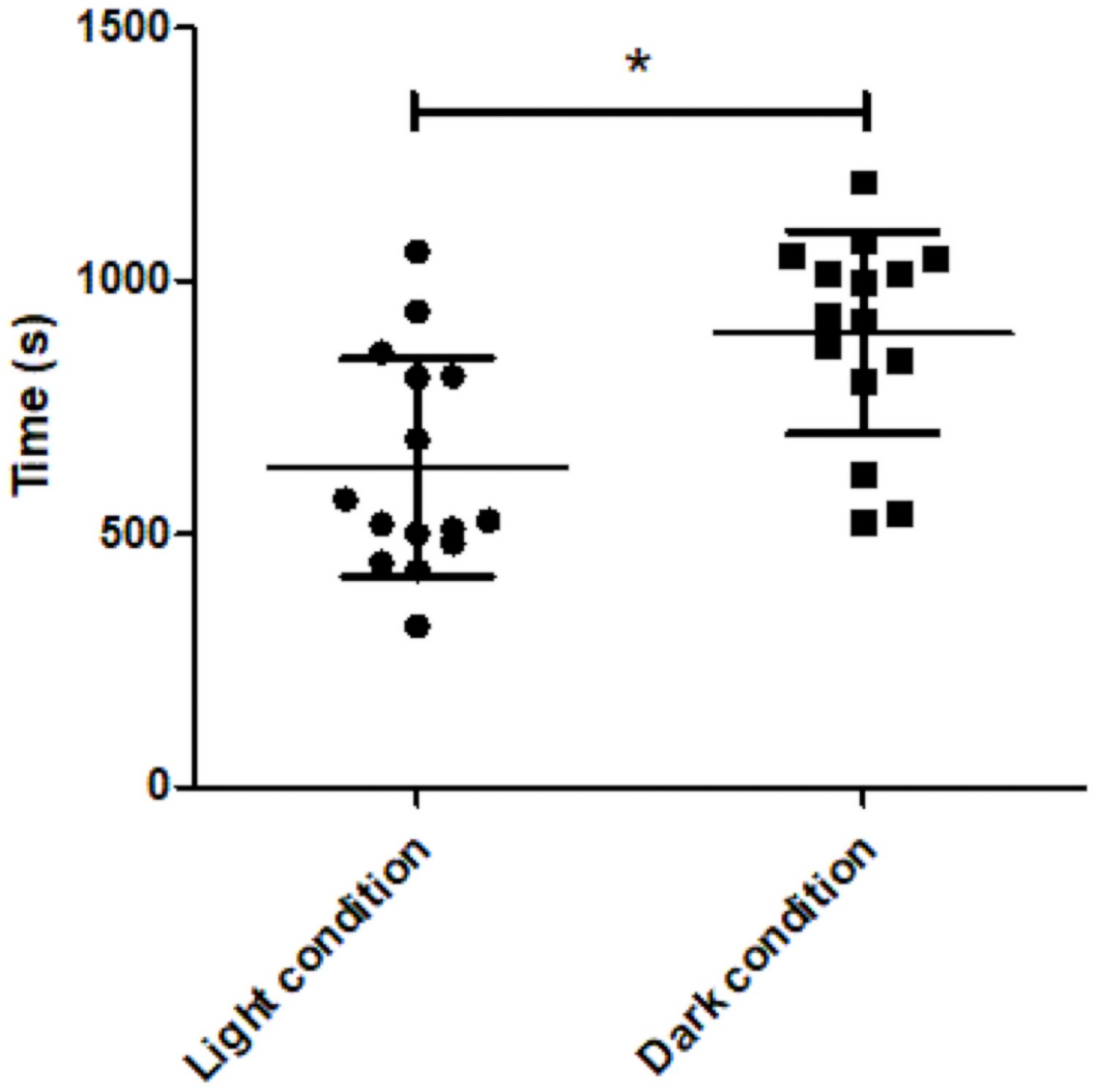
Animals activity. Individual values of the activity recorded over 30 min under light (circles) and dark (squares) conditions. Statistically significant differences are found between these two groups (*p<0.05).

We also found a difference in motor activity between the two conditions (Fig 3). The results showed a significant increase in activity when animals were in dark conditions (898 ± 200 s, p = 0.01) compared to light conditions (632 ± 215 s). We found no difference in activity between the sexes (p = 0.416). Moreover, we found no interaction between animal sex and the illumination condition (p = 0.314).

## Discussion

In this study, we evaluated the effect of ambient light conditions on brain [18F]FDG uptake in mice. We found an increase in [18F]FDG uptake in several brain areas, including the nucleus accumbens, hippocampus, midbrain, hindbrain, and cerebellum, when the mice were placed in dark, compared to light conditions. We also observed that animals showed increased motor activity in dark, compared to light conditions.

In rodents, the nucleus accumbens is involved in the dopaminergic system. This system has been related to attention, memory [14], and wake/sleep regulation [15], among other functions. The hippocampus is related to spatial recognition and spatial memory [16, 17]. The midbrain includes the superior colliculus, in which visual, auditory, and somatosensory information is integrated to initiate motor commands [18]. The midbrain also includes the reticular nucleus, which regulates wake/sleep states [19]. The hindbrain regulates motor, sensory, and visceral functions [20]. Finally, the cerebellum mainly coordinates motor functions [20]. All these structures play similar roles in mice and humans [21].

It is well known that an increase in brain activity induces higher brain glucose metabolism, which we measured as [18F]FDG brain uptake [22]. Refinetti et al. [23] showed that rodents were more active in darkness, due to their nocturnal lifestyle. During the night, rodents perform most of their daily living activities, such as environmental recognition, foraging, feeding, alertness, etc. [24], which are associated with increases in brain activity in certain brain areas, including the nucleus accumbens, hippocampus, and midbrain [14, 18, 19, 25]. Thus, our finding that [18F]FDG uptake increased in certain brain areas in dark conditions could be related to the more intense motor activity observed in our video recordings in darkness. Nevertheless, other factors, such as stress or hormone levels [26, 27], can also affect glucose uptake. We hypothesized that the light/dark condition induced a behavioural change in the animals that led to increased [18F]FDG uptake in the nucleus accumbens, hippocampus, midbrain, hindbrain, and cerebellum. The existence of a sex*illumination interaction might point to a sex-related differential in behaviour, although the effect size of this interaction was nearly negligible (about 40–50 voxels volume, 5–6 mm^3^), compared to the increase in uptake observed in the dark condition (600 voxels volume, 70 mm^3^). The effects found in our study are not very strong (<5%) but they are in line with size effects commonly reported in scientific literature using SPM in mice studies [28, 29].

Many factors that can alter [18F]FDG uptake in rodents [7, 9] are well defined in the literature. However, ambient illumination is not typically reported in preclinical research studies, despite the fact that, as we have shown, it can affect the [18F]FDG brain biodistribution. Therefore, changes in ambient lighting might alter study results.

Our findings had several limitations. First, the animals used for PET imaging were different from those involved in the assessment of motor activity. Thus, we could not observe a direct correlation between the degree of motor activity and the amount of brain uptake. Another limitation was that we did not test hormone levels or stress indicators that might have affected the [18F]FDG brain uptake [3, 26]. Moreover, we could not rule out the possibility that the animals might have been affected by other stimuli, such as noise or odours, which were not specifically controlled during the uptake or the recording period. Regarding multiple comparisons, the voxel-wise [18F]FDG-PET imaging analyses were not corrected for them at voxel level due to the impossibility of assuming independence between adjacent voxels. Instead, in order to control for type II error only significant regions larger than 25 activated connected voxels were accepted, and a cluster-based FWE (family-wise error) correction was applied [30]. Finally, we only studied a single rodent species and strain; therefore, our findings might not be generalizable to all rodent species and strains.

## Conclusions

In conclusion, we objectively assessed how room illumination conditions during [18F]FDG uptake affected the brain [18F]FDG biodistribution. We found that darkness induced an increase in [18F]FDG uptake in the nucleus accumbens, hippocampus, midbrain, hindbrain, and cerebellum. This finding pointed out the importance of controlling and reporting ambient illumination conditions in experiments that involve [18F]FDG uptake. Thus, ambient lighting, along with other common factors (fasting, temperature, etc.), should be controlled in rodent brain [18F]FDG-PET studies to provide more reliable and reproducible results.
